# Supplementary material for: Self-organizing maps of typhoon tracks allow for flood forecasts up to two days in advance
Source: Nat Commun. 2020 Apr 24;11:1983. doi: 10.1038/s41467-020-15734-7 (PMC7181664; doi:10.1038/s41467-020-15734-7)
Supplement: Supplementary file 1 — Supplementary Info [file 41467_2020_15734_MOESM1_ESM.pdf]

SUPPLEMENTARY INFORMATION

Self-organizing maps of typhoon tracks allow for flood forecasts up to two days in advance by Chang, et al.

Supplementary Figures

Supplementary Figure 1 | 87 typhoon tracks that moved across Taiwan between 1965 through 2015

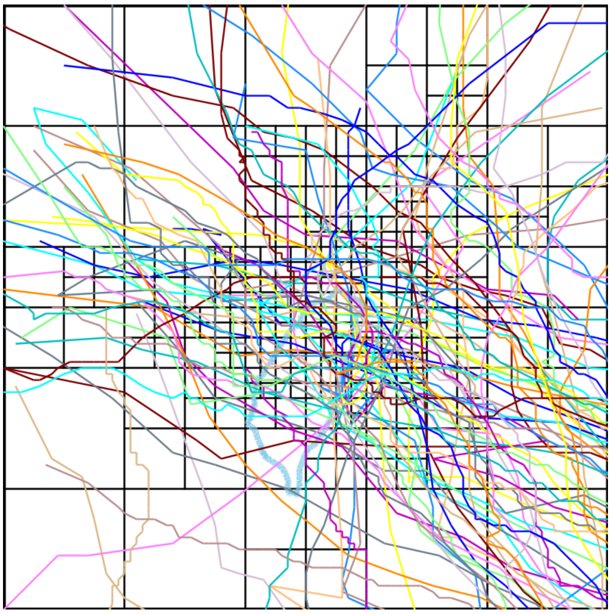

Supplementary Figure 2 | Goodness-of-fit of AI-based and SFM approaches for Typhoon Lekima.

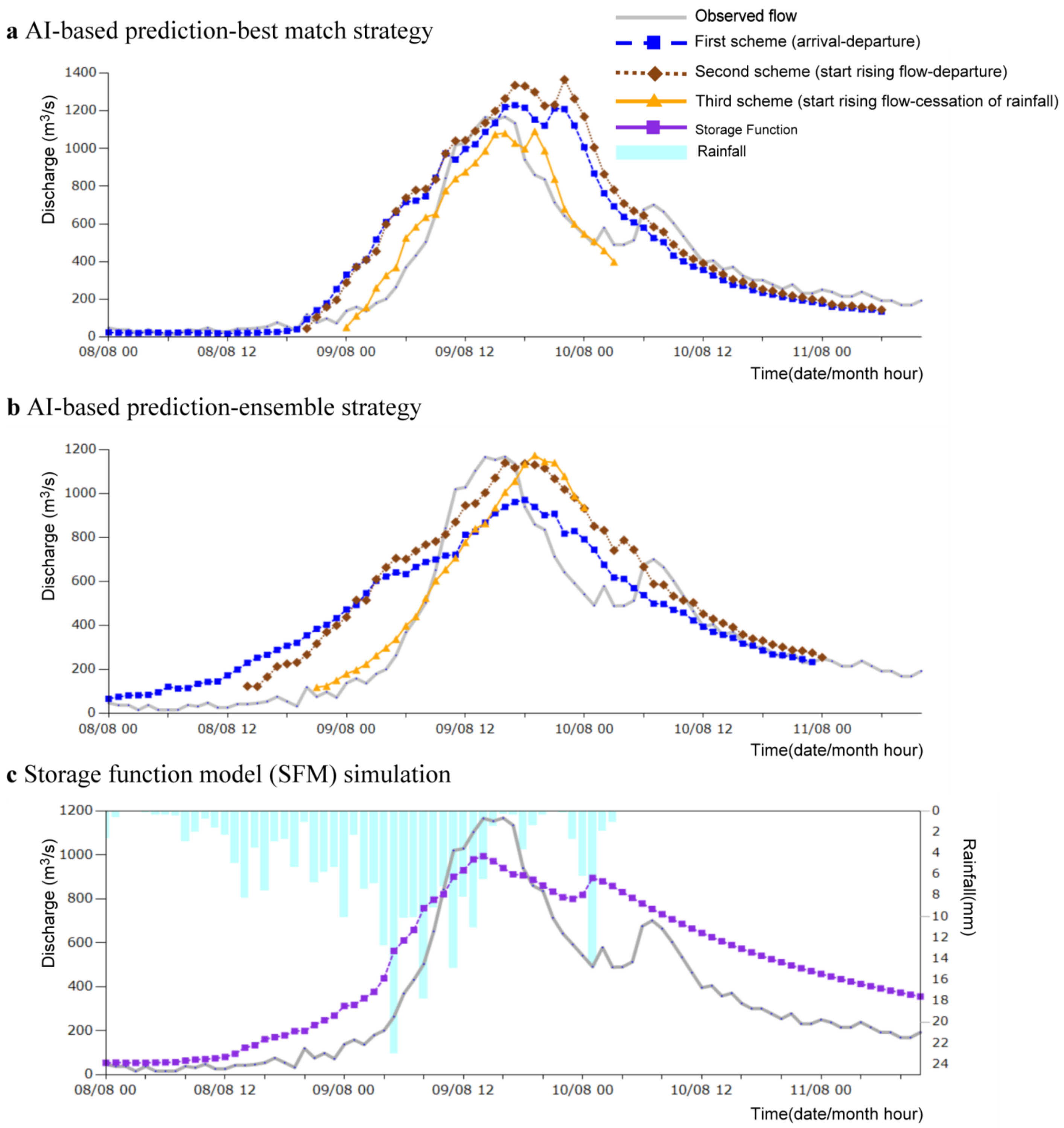

AI-based predictions use two flow characteristic curve selection strategies and three typhoon duration schemes. (a) Predicted flood hydrographs using the first selection strategy, which selects the flow characteristic curve of the best matched track. (b) Predicted flood hydrographs using the second selection strategy, which selects the ensemble of all low characteristic curves in the best matched cluster. We use three schemes to characterize typhoon duration. The first scheme spans between the arrival and departure time of a typhoon over the gridded zone. The second scheme spans between the start rising flow limb characteristics and the departure of the typhoon from the gridded zone. The third scheme spans between the start rising limb characteristics and the cessation of rainfall.

Supplementary Figure 3 | Goodness-of-fit of AI-based and SFM approaches for Typhoon Aere.

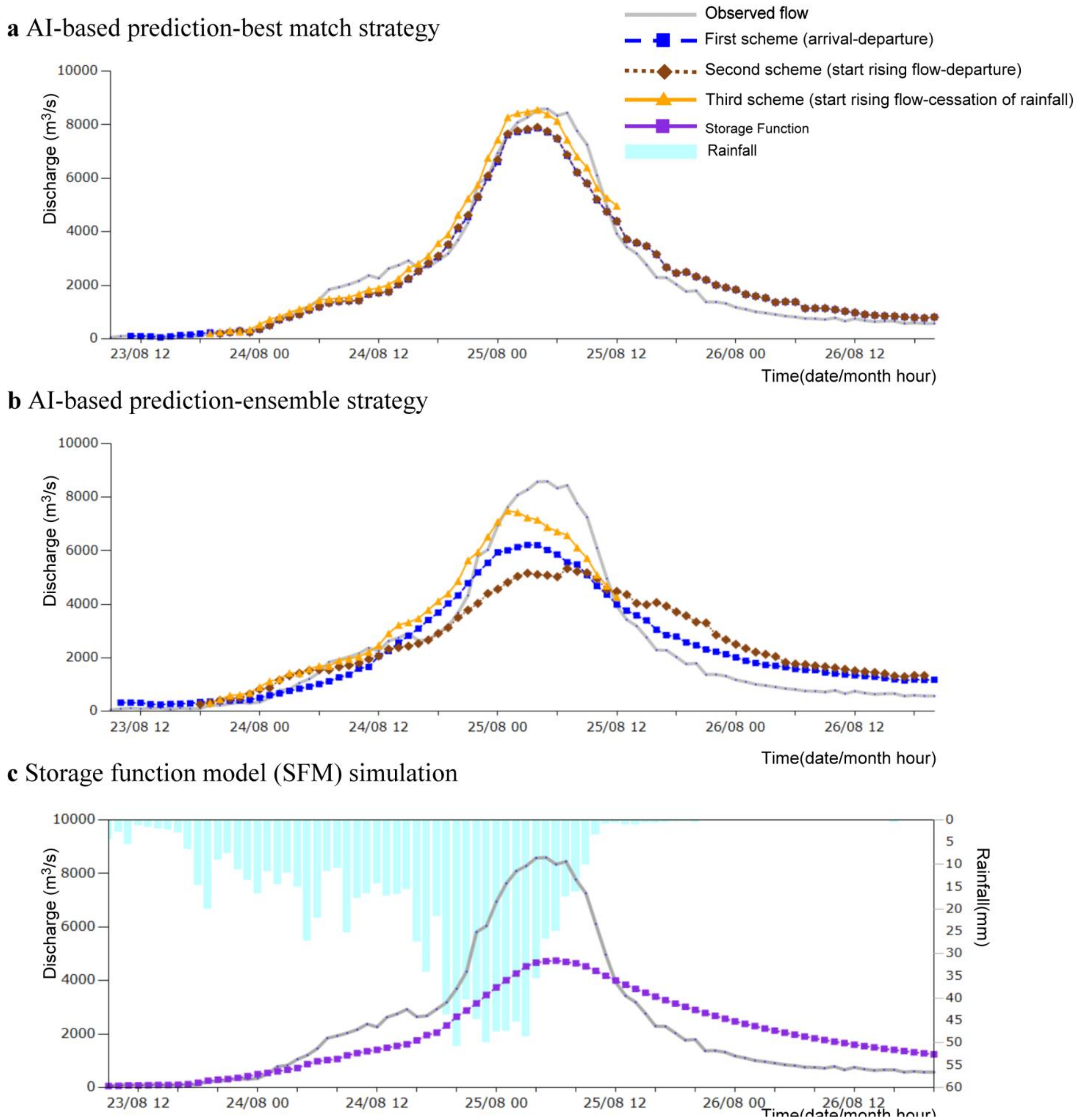

AI-based predictions use two flow characteristic curve selection strategies and three typhoon duration schemes.

Supplementary Figure 4 | Predicted flood hydrographs for the real track of Typhoon Lekima.

a Typhoon tracks

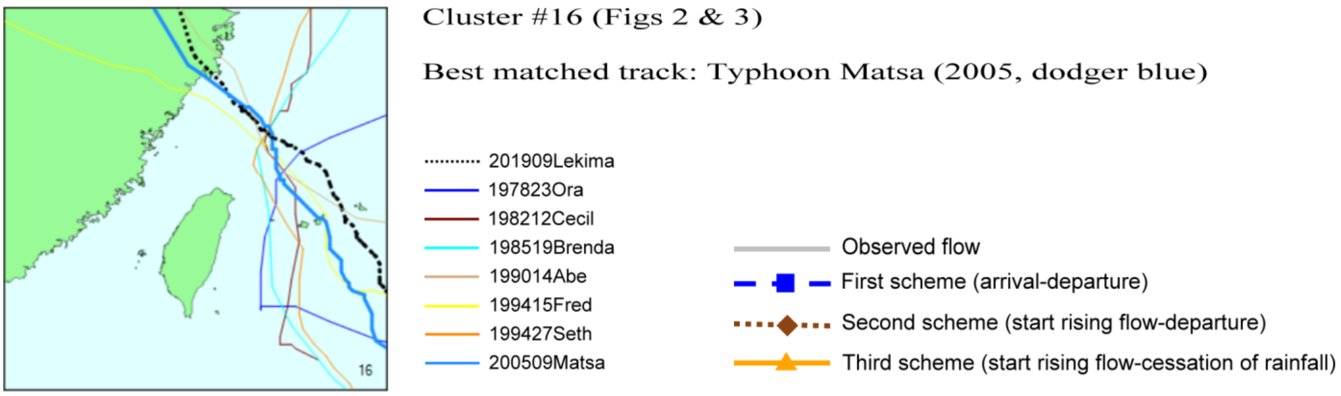

b FCC selection strategy 1

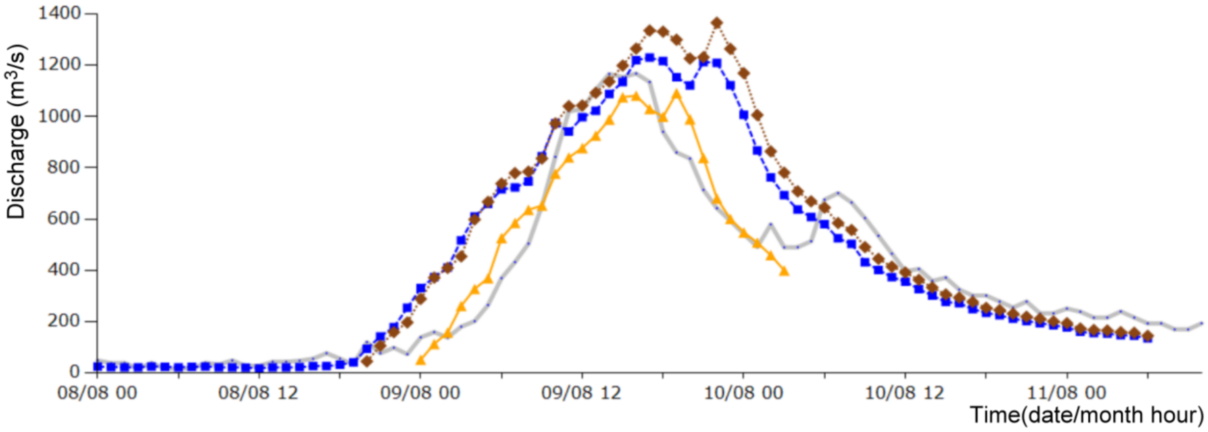

c FCC selection strategy 2

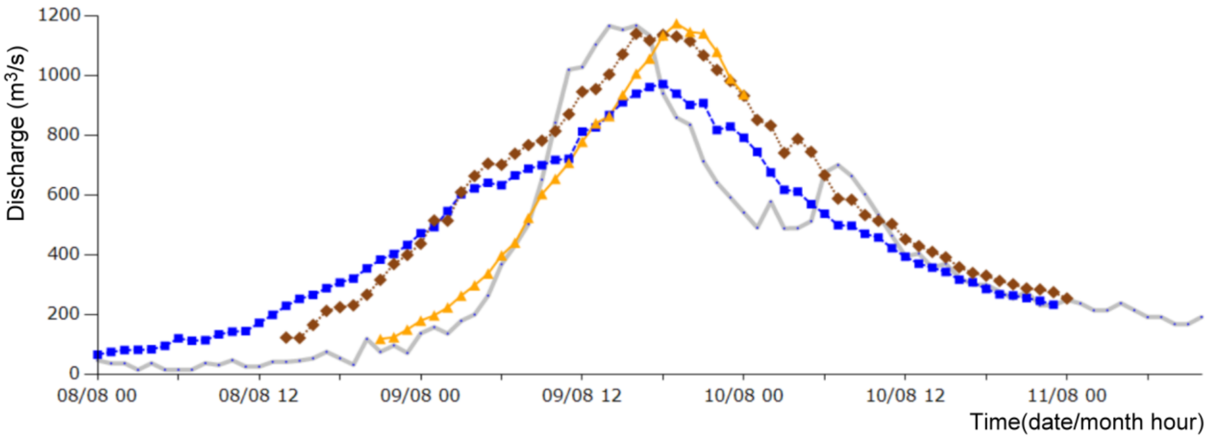

Predictions were made using two flow characteristic curve selection strategies under three typhoon duration schemes. (a) Actual typhoon tracks grouped in cluster (neuron) #16. This is the best matched cluster while Typhoon Matsa is the best matched track. (b) Predicted flood hydrographs using the first selection strategy. (c) Predicted flood hydrographs using the second selection strategy.

Supplementary Figure 5 | Predicted flood hydrographs for the predicted track of Typhoon Lekima

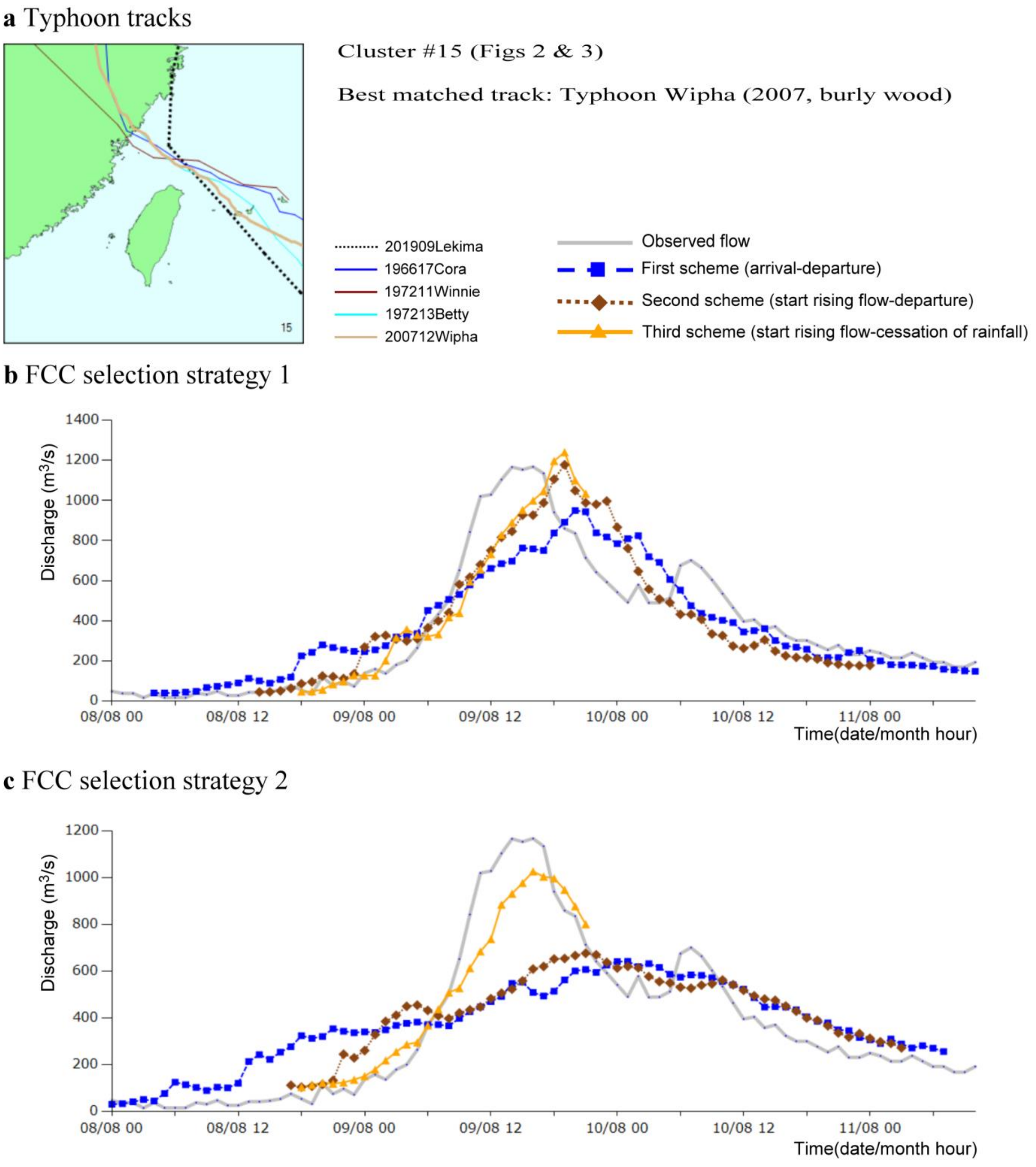

Predicted track: Inaccurate predicted track (8/7 4:00). Predictions were made using two flow characteristic curve selection strategies under three typhoon duration schemes. (a) Actual typhoon tracks grouped in cluster (neuron) #15. This is the best matched cluster while Typhoon Wipha is the best matched track. (b) Predicted flood hydrographs using the first selection strategy. (c) Predicted flood hydrographs using the second selection strategy.

Supplementary Table

Supplementary Table 1 | Test results of ten typhoon events using (1) AI-based models under three TD schemes with two FCC strategies and (2) SFM model

Typhoon	Arrival-departure[1]				Rising-departure[2]				Rising-cessation[3]				Storage Function Model[6] (SFM)	
	Best Match[4]		Average[5]		Best Match		Average		Best Match		Average			
	RMSE	R^2	RMSE	R^2	RMSE	R^2	RMSE	R^2	RMSE	R^2	RMSE	R^2	RMSE	R^2
198119Clara[7]	185	0.68	125	0.76	179	0.71	144	0.81	212	0.68	139	0.95	209	0.86
200116Nari	707	0.61	869	0.35	761	0.60	1074	0.07	871	0.42	943	0.31	578	0.74
201307Soulik	782	0.82	775	0.84	632	0.86	1126	0.51	458	0.96	543	0.96	1086	0.65
201323Fitow	262	0.91	254	0.59	95	0.95	230	0.80	157	0.94	112	0.96	205	0.76
201416Fung-Wong	54	0.53	53	0.58	70	0.48	72	0.43	51	0.98	67	0.91	106	0.61
201521Dujuan	141	0.98	327	0.93	144	0.98	238	0.95	231	0.99	582	0.83	583	0.70
201616Malakas	112	0.93	138	0.92	140	0.76	177	0.51	118	0.91	124	0.93	78	0.96
201709Nesat	92	0.73	114	0.57	104	0.80	129	0.60	24	0.95	24	0.99	158	0.55
201808Maria	184	0.85	365	0.33	144	0.91	215	0.86	287	0.92	202	0.89	250	0.82
201909Lekima	170	0.83	171	0.81	202	0.76	185	0.68	116	0.89	222	0.69	162	0.83
2019/8/7 04:00	165	0.74	241	0.47	179	0.73	248	0.45	192	0.80	127	0.93		
2019/8/6 04:00	174	0.79	175	0.75	204	0.70	247	0.44	228	0.73	253	0.58		
200417Aere	515	0.96	967	0.89	542	0.96	1390	0.77	496	0.97	870	0.92	1425	0.74

[1] The arrival and departure time of a typhoon over the gridded zone

[2] The rising limb characteristics related to the departure of the typhoon from the gridded zone

[3] The rising limb characteristics related to the cessation of rainfall

[4] The FCC of the best matched typhoon track

[5] The average of all FCCs in this matched neuron

[6] The simulation results by using SFM model

[7] The first four digits denote the year followed by the two-digit serial number in the year of a typhoon
